# HIV Treatment as Prevention: Models, Data, and Questions—Towards Evidence-Based Decision-Making

**DOI:** 10.1371/journal.pmed.1001259

**Published:** 2012-07-10

**Authors:** The HIV Modelling Consortium Treatment as Prevention Editorial Writing Group

**Affiliations:** Duke University Medical Center, United States of America

## Abstract

Antiretroviral therapy (ART) for those infected with HIV can prevent onward transmission of infection, but biological efficacy alone is not enough to guide policy decisions about the role of ART in reducing HIV incidence. Epidemiology, economics, demography, statistics, biology, and mathematical modelling will be central in framing key decisions in the optimal use of ART. *PLoS Medicine,* with the HIV Modelling Consortium, has commissioned a set of articles that examine different aspects of HIV treatment as prevention with a forward-looking research agenda. Interlocking themes across these articles are discussed in this introduction. We hope that this article, and others in the collection, will provide a foundation upon which greater collaborations between disciplines will be formed, and will afford deeper insights into the key factors involved, to help strengthen the support for evidence-based decision-making in HIV prevention.

## Introduction

The 19th International AIDS Conference will meet in Washington, District of Columbia, 22–27 July 2012. Since the last International AIDS Conference in Vienna two years ago, more than five million people globally have become newly infected with HIV [1],[2]. In South Africa, a country with one of the largest HIV epidemics, 3% of the young men and women who were 19 years old and uninfected at the time of the last conference will now be infected [3]. Indications that the rate of new HIV infections in several countries may have declined recently are extremely welcome. Moreover, the recent UNAIDS Investment Framework [4] and President's Emergency Plan for AIDS Relief guidance on combination prevention [5] suggest that combining existing interventions and scaling them up could have further significant impact on reducing HIV incidence. However, these strategies are not expected to bring the epidemic fully under control.

Advances in HIV prevention research over the past two years have generated considerable optimism. First, it was shown that a 1% tenofovir vaginal microbicide gel reduced HIV acquisition in women in South Africa [6], and this was followed by a trial demonstrating that daily oral co-formulated tenofovir and emtricitabine reduced the risk of HIV acquisition in men who have sex with men (MSM) [7]. Subsequently, daily oral tenofovir alone or combined with emtricitabine was shown to reduce the risk of HIV acquisition in heterosexual men and women in long-term relationships in Uganda and Kenya [8]. There have also been some indications that a vaccine candidate (RV144) provides some short-term protection against infection [9]. These modalities provide a partial reduction in risk, but some studies on pre-exposure prophylaxis have produced conflicting results, highlighting that many questions in this field remain unanswered [10].

However, the finding that has created that greatest excitement has been that HIV-infected individuals who are given antiretroviral therapy (ART) are much less likely to transmit the infection to their heterosexual partners than those who are not. This finding was shown in the HPTN 052 trial [11] (Box 1), which was chosen as the *Science* magazine breakthrough of the year for 2011 [12]. If viral load is fully suppressed, those on ART may effectively be almost uninfectious. Although anticipated [13],[14], this finding has catalyzed enormous interest in how ART could not only benefit the individual provided with the medicines, but also reduce the epidemic burden of the communities in which they live by limiting HIV transmission.

**Box 1. The HPTN 052 Trial**

The HPTN 052 trial enrolled 1,763 HIV-1 serodiscordant couples (i.e., couples in which one partner is HIV-infected but the other is not) in which the CD4 cell count for the HIV-infected partner was between 350 and 550 cells/µl. The HIV-infected partners were randomized either to receive ART immediately (“early ART” arm) or to receive ART when their CD4 cell count dropped below 250 cells/µl (“delayed ART” arm). The couples were followed up for a median of 1.7 years, and substantial effort was made to ensure that viral suppression was achieved among those in the early ART arm. A total of 39 transmission events were observed. Genetic linkage analysis confirmed that 28 of these were linked to the stable partner. Of these 28 linked transmissions, 27 were in the delayed ART arm and one was in the early ART arm, resulting in an estimated 96% reduction (95% confidence interval: 73%–99%) in the risk of transmission from HIV-infected individuals on early ART compared with delayed ART. Earlier ART was also associated with significant improvement in a composite indicator of morbidity and mortality (41% [95% confidence interval: 12%–60%] reduction).

Although the HPTN 052 study was the first randomized controlled study to demonstrate the impact of ART on transmission, an earlier observational study among couples recruited for another trial had previously indicated that ART was associated with a 92% reduction in the risk of transmission [13]. Other observational studies also support that the risk of transmission when virally suppressed on ART is very substantially reduced [14],[57]. However, many questions remain about the impact of ART on transmission, including the durability of the effect, levels of suppression that would be possible in other settings, and the impact through other routes of HIV transmission (especially unprotected anal sex).

The role of ART in reducing HIV incidence will probably be among the most important topics in the field of HIV prevention for years to come, and it is already being debated urgently at national and international levels, within major normative agencies and charities, and by donors and implementers. The issues cut across the domains of epidemiology, economics, statistics, demography, virology and immunology, behavioural science, mathematical modelling, and clinical trials, and demand an interdisciplinary approach.

The HIV Modelling Consortium aims to coordinate and promote research across these disciplines and streamline communication between decision-makers and researchers. Mathematical modellers have considered the potential impact of ART on HIV incidence in a variety of scenarios and settings over the past 15 years, with model estimates becoming more refined as improved data have become available. A collaborative meeting of the HIV Modelling Consortium was held in November 2011 (http://www.hivmodelling.org/events/potential-impact-treatment-hiv-incidence) to review findings and identify priorities for future research. Several interlocking themes arose at the meeting, which are covered by the set of articles in this special collection, “Investigating the Impact of Treatment on New HIV Infections” (http://www.ploscollections.org/TasP2012) [15]–[23]. In this article we seek to set each piece in context, and describe important issues that are beyond the scope of this collection.

## The Potential Impact of ART on HIV Incidence

**Figure 1:**
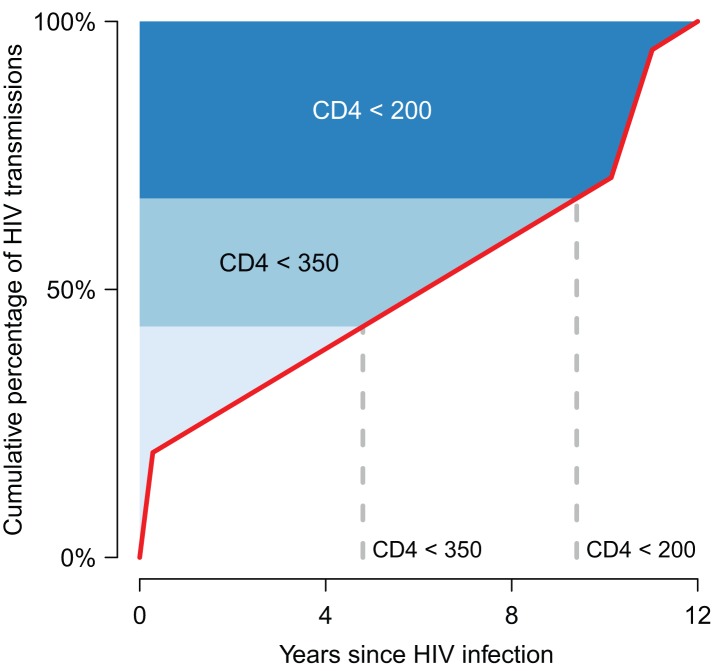
A framework for understanding the epidemiological impact of HIV treatment. The published results of models [38],[53]–[55] that have estimated the contribution of different stages of HIV infection to onward transmission are compiled in a median cumulative distribution of infections generated by one infected person over the course of his/her infection in the absence of treatment (red line). The horizontal axis shows time from the time of infection to 12 years, which is the mean survival time for those with untreated HIV infection [56]. The vertical axis shows the cumulative transmission, from 0% (no new infections generated yet) to 100% (all onward transmission completed). (Note that the uncertainty in this distribution is not indicated.) The shading indicates the approximate CD4 cell count category at each time point [25],[26]. Currently, treatment tends to be initiated well below a CD4 cell count of 200 cells/μl [32], meaning that the contribution of treatment to prevention is minimal because most of the transmission from that person has already occurred before treatment starts. If increased testing and improved linkages to care enabled individuals to start treatment at a CD4 cell count very close to 200 cells/μl, this could result in a substantial reduction in HIV incidence, because ∼25%–30% of transmission normally arises from individuals after that point. The prevention impact would be expected to be even greater with initiation close to a CD4 cell count of 350 cells/μl. If the average number of new infections arising from an infected person in a susceptible population exceeds one before treatment could be feasibility initiated, then treatment could not eliminate the HIV epidemic. In this framework, the influence of other forms of prevention will be to change the shape of the graph. For instance, if many men are circumcised or individuals have fewer new sexual partners per time unit, then new infections arising from an infected person will grow more slowly over time, so that on average one new infection might be generated only after the point at which a feasible programme could have initiated treatment.

Fundamentally, the impact that a treatment programme can have on preventing infections in an epidemic is determined by two main factors. First, it is determined by the number of onward transmissions generated by a newly infected person *before* they start treatment, which is determined by the biology of HIV infection, patterns of sexual contact between partners, the effects of other prevention interventions, and the rates of HIV testing and linking to care (Figure 1). Second, the impact is determined by the number of onward infections generated by an individual *after* ART initiation, which additionally depends on the biological efficacy of treatment, as well as adherence and retention in care. Estimating the population-level impact of expanded access to ART therefore involves synthesising diverse sources of information and managing substantial amounts of uncertainty about virology, immunology, human sexual behaviour, and the long-term performance of prevention programmes. The biological efficacy data provided by the HPTN 052 trial [11] is only one piece of this puzzle.

Mathematical models provide a framework within which to assemble this information, and several models of the epicentre of the worldwide epidemic, sub-Saharan Africa, have been developed and used to investigate the potential impact of treatment on HIV incidence. As different studies have addressed different questions and made different assumptions, it has been unclear whether or not these models fundamentally agree about the potential impact of particular treatment interventions in reducing HIV incidence. If they do, this could increase confidence in their collective findings, but if they do not, then this provides an important note of caution when considering results and highlights areas for further investigation.

### A Systematic Comparison of 12 Models

In this collection, Eaton et al. [15] present the results of a systematic model comparison exercise in which 12 of these models were used to simulate the same sets of interventions. The model results were relatively consistent for short-term (eight-year) projections of reductions in incidence associated with treatment. For instance, if, hypothetically, 80% of individuals were treated after their CD4 cell count reaches 350 cells/μl (approximating current international guidelines; Box 2), the models projected that the incidence rate would be reduced by 35%–54% after eight years, compared with what the incidence would be in the absence of any ART. All models suggested that the existing treatment scale-up in South Africa should have already reduced new infections (incidence in 2011 is estimated to be 17%–32% lower than if there had been no ART [15]). The consensus that treatment provided within current guidelines has a prevention benefit is significant and should serve to reinforce the case for continuing to improve access to ART. However, there was much more variation in long-term (38-year) projections of reductions in incidence. One important way in which the models differ is in how they represent the behaviours leading to transmission, such as heterogeneity in sexual risk behaviours and patterns of contact with respect to age, which are notoriously hard to quantify [24]. Another difference is in how they represent the biology of infection, in particular the rate of CD4 cell count decline and relative infectiousness [25],[26], about which there is little comprehensive agreement. It will be important to consider the influence of these factors on the key outcomes of interest when interpreting future modelling studies on this topic.

**Box 2. Current International Guidelines for Use of ART**

The current World Health Organization international guidance recommends that HIV-infected patients with CD4 cell count ≤350 cells/µl be initiated on ART. In addition, patients with advanced clinical disease or HIV-infected people with active tuberculosis should be immediately initiated on ART, irrespective of CD4 cell count [58]. In April 2012, new guidance was issued that HIV-infected individuals with a long-term partner who is HIV-uninfected could also be considered for ART initiation [59]. New guidelines will be promulgated by the World Health Organization in 2013 [60].

However, these guidelines do not necessarily reflect the care that patients actually receive. National guidelines may or may not fully reflect the World Health Organization guidance, and typically, constraints on resources, the capacity of health systems, and care-seeking behavior result in individuals being initiated on ART at lower CD4 cell counts than the guidelines recommend.

### Connecting Model Projections to the “Real World”

When using extremely ambitious assumptions about the ability of ART programmes to test and start treatment of HIV-infected individuals very soon after infection, and retain them in care, five of nine models compared by Eaton et al. [15] suggested that incidence would be reduced by more than 90%, similar to the modelling predictions reported by Granich et al. [27]. However, these assumptions can be contrasted with recent real world experience in which the HIV testing rate was 52% in the cross-sectional, nationally representative South African National HIV Prevalence, Incidence, Behaviour and Communication Survey [3], and the repeat testing rate of individuals in an intensive community-mobilising intervention was 28% [28]. In addition, linkage from testing to ART uptake is assumed to be 100% in the models, but has been about 33% in actual programmes [29]. Rather than 0% refusal of uptake of treatment, as assumed in the models, some settings have seen 20% refusal [30]. Finally, the dropout rate from programmes was 1.7% per year in the most optimistic model simulations presented in Eaton et al., compared with around 10% over the first year in the IeDEA network of clinics [31]–[33].

These inconsistencies between modelling assumptions and projects and real world situations do not mean that treatment cannot be used to generate greater reductions in incidence, but rather that major advances in programme coverage and delivery will be required to fully exploit the potential prevention benefits of treatment. These are operational barriers that could be improved without the development of new scientific prevention technologies, but which will nevertheless require substantial investment in health services.

In many models, including several of those in the modelling comparison [15], several significant simplifying assumptions about other factors that might influence success were made, because the exercise was focussed on the impact of a simple and stylized treatment programme on HIV incidence. In particular, most models did not explicitly include the relationship between adherence to ART regimens and degree of viral suppression, which would affect the therapeutic benefit, the prevention effect, and the potential for emergence of drug-resistant virus. Drug resistance is an important issue, especially over the long timescales considered here, because it effectively weakens the impact of existing first-line regimens and could cause greater reliance on second- and third-line treatment regimens, which are currently more expensive. There are many other considerations that the modelling comparison by Eaton et al. did not address, such as the interaction of ART with behavioural interventions and the best use of diagnostic tools that could measure viral load or CD4 cell count at point of care, which are also the subject of ongoing research [34]–[37] but beyond the scope of this collection.

### Evidence of Impact from Existing Programmes

Consensus across multiple models can be reassuring, but it is still possible that all the models could be wrong if, for instance, the small number of key data sources they rely on are not representative, or if all the models do not incorporate some crucial aspects of the system. Another essential check for models is a comparison of their projections with real data: in this case, the observed impact of treatment programmes in industrialised countries that have already achieved good access to treatment [38]. In this collection, Smith et al. [16] review the data that have been interpreted as showing that treatment has already had an impact on reducing incidence, showing apparent consistency between modelled expectations and reality. However, Smith et al.[16] advise caution when interpreting the level of evidence implied, particularly where indirect metrics for ART exposure (such as community viral load) and proxies for HIV incidence (such as new diagnoses) are used.

In this collection, Wilson [17] describes the examples of Australia and France, among other settings, where, despite high testing rates and coverage of treatment among MSM, HIV incidence has not decreased. This is in contrast to what models suggest should have occurred if the assumptions about treatment as prevention from heterosexual studies are applied to MSM populations. It will be essential for modellers to learn from the past by reconciling these and other observations to refine future model projections.

### The Role of Early HIV Infection

One particular issue that may prevent even the most ambitious treatment programmes from reducing HIV epidemics to very low levels is the role of early HIV infection in sustaining HIV transmission. Early HIV infection covers the time shortly after infection—and usually before HIV diagnosis—when viral concentration in the blood spikes and individuals are more infectious [39]. If a substantial proportion of transmission occurs during early infection, the impact of treatment programmes will be less [40] (Figure 1). This could in part explain the apparent lack of preventive efficacy of ART in epidemics among MSM, as explored in Australia and elsewhere by Wilson [17] and in other examples examined by Kumi Smith et al. [16]. However, it is an open question whether early HIV infection is a dominant factor in sustaining epidemics in sub-Saharan Africa, and it has been argued that the contribution of acute infection to sustaining epidemics is not a primary determinant of the impact of treatment interventions. In this collection, Cohen et al. [18] debate the size and significance of this effect, and call for new data to be collected that may help this to be determined.

## Economic Considerations

Ideally, public health policy should be driven by maximising improvements in the health of populations, rather than by economic considerations. But the HPTN 052 [11] findings have come at a difficult time for the public health response to HIV. After years of rapid growth, funding commitments and disbursements have stabilised or been reduced [41], and only a few countries in sub-Saharan Africa are currently able to achieve the high levels of treatment coverage for those eligible recommended by current international guidelines (Box 2) [1],[2]. While the cost of providing treatment has fallen dramatically in recent years [42], offering ART to individuals who are not in immediate clinical need may continue to be significantly more expensive and complex than other existing methods for reducing HIV transmission, such as male circumcision [43] and some forms of behaviour change communication interventions (in particular, voluntary counselling and testing) [44]–[46].

To some policy-makers, the slowdown of growth in budgets available for HIV/AIDS programmes is a sobering constraint and makes the potential benefits of radical programmes with high near-term costs irrelevant. Their questions are about the most cost-effective allocation of incremental changes in resources and portfolio optimisation in light of the new data about the additional effect of reducing new infections. To others, the squeeze on funding is a cue to look for ways to drive large reductions in the need for resources in the future, which could be generated by an overhaul of the current epidemic response and an increase in resources in the short term. New, large investments in controlling HIV may not be impossible, but there would have to be a strong case for the return on such an investment.

### Estimating Costs

In this collection, Meyer-Rath and Over [19] outline economic concepts that should guide discussions about the potential for ART to reduce incidence, and how the programmatic targets identified by epidemiological modelling could translate into costs. They argue that the nature of the cost function for ART—that is, the cost of providing additional patient-years of ART given the current scale of a programme and practical constraints—has received insufficient attention in earlier analyses. In particular, they suggest that the scale and scope of a country's ART programme, including clinic size and density, cohort maturity, patient mix, and health-worker effectiveness, could mean that the cost of scale-up of ambitious treatment programmes has been substantially underestimated. However, some projected increases in cost could be offset if future programmes radically change by simplifying the delivery of treatment, such as by eliminating measurement of CD4 counts and/or pre-ART disease monitoring. In a commentary in this collection on the review by Meyer-Rath and Over, Bärnighausen et al. [20] consider the dilemma for those making economic projections for the use of ART as prevention. As ART for prevention represents a significant departure from the current practice of ART for those in greatest clinical need, it is not clear how to extrapolate from past experience.

### Short-Term versus Long-Term Goals

Another key economic consideration is finding the right level of spending now to provide the potential for significant benefits in the long term. The HIV/AIDS epidemic and interventions to stop HIV and AIDS are inevitably long-wave events [47], so this issue is particularly important. Treatment programmes spend money today for returns (in terms of averted infections and deaths, or reduced costs) years and even decades later. If future costs and benefits have the same value as current ones, then enormous sums spent today to eventually avert greater costs and reduce mortality forever (if incidence is reduced to low levels) would be judged as a worthwhile expenditure. Whereas, if we accept that, to a decision-maker, savings that are accrued in the future may be worth less than those made today, then potential future payoffs may be less attractive, and investment in programmes for other, more immediate causes of mortality would be a rational, if not necessarily an inspiring or ethical, response. Recognition of this reality for decision-makers requires modellers to vary the relative value that is assigned to costs and benefits in the future. This is called discounting. Discounting is just one component of how decisions are made, and it is important for those contributing to the debate to be able to couch their arguments in the context of this fundamental consideration.

### Working within Economic Constraints while Increasing Access

Those making decisions about expanding the provision of ART for prevention purposes must also plan for the long-term maintenance of such a commitment. Once treatment is initiated, it is lifelong, and, because relying on treatment to reduce incidence does not inherently alter the underlying drivers of infectious spread (e.g., patterns of sexual contact), future reduction of an ART intervention effort could lead to a resurgence of the epidemic. Given economic constraints, the most likely scenario might be for programmes to increase access to treatment gradually. They could increase access by expanding eligibility criteria incrementally to include groups who are most likely to benefit clinically, and whose treatment will most reduce onward transmission. Several possibilities for doing this have been raised, including prioritisation according to biological characteristics (e.g., pregnant women, those with active tuberculosis, or those with high plasma viral loads) or according to behaviours (those in serodiscordant couples, those attending sexually transmitted infection clinics, those with many sexual partners, or sex workers). The epidemiological benefit of providing increased access to treatment for groups beyond current guidelines will be determined by the extent to which the criteria being used to prioritise individuals can reliably identify those who most need treatment or contribute most to generating new infections.

There are also many other factors that should be considered in prioritising groups for expanded ART. These include the size of the group and affordability. The cost to access the group is another factor. For instance, would it be less costly to reach pregnant women, who are already in contact with the health system, than some other groups? The response of a group to treatment also needs to be considered. For example, would stable couples adhere to treatment better than others, or would adherence be low if there is little immediate therapeutic benefit? Ethical considerations, programme acceptance, and feasibility also need to be taken into account. For instance, would it be acceptable to provide serodiscordant couples with preferential access to treatment? These layers of considerations will not always point to one particular group as the best option, and local epidemic, economic, and social conditions will also influence this choice. In addition to these judgments being unlikely to be clear-cut, they are further complicated in instances in which human rights and public health do not necessarily have the same objectives if followed to their logical ends, for instance, if the best strategy for a population does not give optimal outcomes for all individuals. In this collection, Delva et al. [21] review these issues for a wide set of prioritisation options, and Boily et al. [22] describe how mathematical modelling can be used to design, conduct, and analyse studies so that the impact of some of these options can be tested and compared effectively.

Full accounting of the economic costs and benefits of ART includes potentially significant macroeconomic benefits (development of infrastructure, supply chains, and education, and productivity gains) and social benefits (reduced orphaning and increased family stability and employment) derived from spending on ART programmes, which could also have synergies with, and spillover benefits for, interventions for other diseases [48]. These important economic questions are not addressed in this collection of articles. Nevertheless, incorporating these factors into estimates of the cost-effectiveness of alternative forms of interventions [49] or estimates of optimal resource allocation [50] among the repertoire of antiretroviral-drug-based and non-antiretroviral-drug-based prevention interventions, even while uncertainties remain, is an important area of ongoing and future research to help inform decision-making processes.

## Research Agenda: Upcoming Trials

The findings of the HPTN 052 trial [11] demonstrated the biological efficacy of treatment in reducing infectiousness in heterosexual individuals who receive the best care and monitoring that is possible. The durability of the effect over the long term will be the focus of the next phase of HPTN 052 [51]. The efficacy of ART in reducing infectiousness from anal sex among MSM is being investigated in observational studies, such as the Opposites Attract study in Sydney, Australia (A. Grulich, personal communication).

Meanwhile, the operational questions will centre on how to deliver the services that are required for maximising the impact of treatment on epidemic spread: very high coverage of HIV testing, frequently repeated HIV testing, strong linkage to care, and high retention in care. Many studies that are already underway aim to examine some of these issues [52].

Several large cluster randomized controlled trials that aim to measure the impact of treatment interventions on HIV incidence in whole communities will also be initiated shortly. One of these studies, PopART (HPTN 071) [52], will test the hypothesis that greatly expanded access to treatment, in combination with access to other services including safe medical male circumcision, is feasible and reduces HIV incidence in populations by 60%. The trials will provide an important and direct test of the predictions set out by mathematical models, and models will have a key a role in the design of the studies and the interpretation of findings. In this collection, Boily et al. [22] describe PopART and other upcoming trials, and outline the role of modelling before (in planning and design), during (in monitoring), and after (for interpretation and extrapolation) trials.

## Future Directions: Priorities for Modelling

From consultation with programme leaders, key stakeholders, community members, and funders at the HIV Modelling Consortium meeting in 2011, several themes have emerged as priority issues for further analysis and modelling. First, models need to focus more specifically on the impact of decisions over short time horizons, which are of greater relevance when justifying expenditure, as well as on long-term impacts. Second, models should estimate the impact that programmes may have already had and the benefits of current policy decisions, rather than only what impact radically different programmes might have in the distant future. Third, models should estimate impact in a greater variety of settings, including concentrated epidemics. Fourth, models should become better aligned with the experience of real programmes, rather than using unrealistically optimistic assumptions. And, finally, models should be utilised to further explore potential negative outcomes of expanded treatment programmes, such as imperfect adherence, drug resistance, difficulties in recognising resistance if treatment is initiated at high CD4 counts, and the potential influence of compensatory changes in risk behaviours.

At the HIV Modelling Consortium meeting, many called for modelling to articulate the consequences of reductions in funding, such as numbers of new treatment initiations decreasing substantially or even current cohorts of treated patients not being maintained. This modelling would highlight the ethical choices at the heart of these issues. It was also agreed that models should incorporate more fully the benefits of earlier treatment initiation, in terms of a potential, but not certain, additional therapeutic benefit, reduced incidence of tuberculosis, reduced costs of prevention of mother-to-child transmission services, reduced cost of monitoring patients in care and treating opportunistic infections, and spillover effects such as greater productivity and reduced numbers of orphans.

There has also been a call to improve communication of mathematical modelling research to policy-makers, clinicians, and other researchers so as to better integrate its role into the wider scientific process and to more clearly articulate the strengths and weaknesses of particular modelling analyses. In response, Delva et al. [23] in this collection present some principles of “best practice” for model presentation and interpretation, which they hope will become a shared resource for both those who conduct modelling research and those who use modelling results.

## Conclusions

The question of how to best use the tools that have been shown to reduce HIV transmission will likely dominate the field of HIV prevention for the foreseeable future. It touches every other aspect of the response to the worldwide HIV epidemics, from the optimal allocation of resources in real programmes, to the relative value of investing in developing additional prevention modalities, to the global spending that will be required in the future. Epidemiology, economics, demography, statistics, and mathematical modelling will be central, and it is hoped that this collection of articles will provide a solid foundation upon which greater collaborations and deeper insights will be formed, and will strengthen the support for evidence-based decision-making, to the benefit of all those whose lives are threatened by HIV epidemics.

**Key Points**

- It has been established that ART for those infected with HIV can prevent onward transmission of infection, but biological efficacy alone is not enough to confirm the impact that ART could have on the HIV epidemic, or to show how best to use ART to reduce incidence of HIV. This will be among the most important issues in the field of HIV prevention for the foreseeable future.
- Epidemiology, economics, demography, statistics, biology, and mathematical modelling will be central in framing key decisions in the optimal use of ART.
- The HIV Modelling Consortium aims to coordinate and promote research across these disciplines, and facilitate communication between researchers and policy-makers. At a collaborative meeting of this consortium in November 2011, several interlocking themes emerged that are discussed in this article and covered in more depth by other articles in this collection.
- Mathematical modelling is used to investigate the potential impact of treatment on HIV incidence. However, because of incomplete information on all the factors that could influence impact, substantial uncertainties will remain. Models should acknowledge those uncertainties and help prioritise data collection where this could strengthen model conclusions.
- The current economic constraints on HIV prevention bring to the fore the role of modelling to help assess the value and cost-effectiveness of ART. Understanding costs and integrating costing and epidemiological models will be key areas of ongoing and future research to help inform decision-making processes. Models are also being used to help design and interpret trials that test hypotheses about the impact of expanded access to treatment on the spread of HIV in communities.
- We hope that this article and others in the collection will provide a solid foundation upon which greater collaborations between disciplines will be formed, so as to better integrate the role of modelling into the wider scientific process and to more clearly articulate the strengths and weaknesses of particular modelling analyses. This approach will ultimately strengthen the support for evidence-based decision-making in HIV programmes.

## Abbreviations

ART: antiretroviral therapy
MSM: men who have sex with men
